# Telmisartan use and risk of dementia in type 2 diabetes patients with hypertension: A population-based cohort study

**DOI:** 10.1371/journal.pmed.1003707

**Published:** 2021-07-19

**Authors:** Chi-Hung Liu, Pi-Shan Sung, Yan-Rong Li, Wen-Kuan Huang, Tay-Wey Lee, Chin-Chang Huang, Tsong-Hai Lee, Tien-Hsing Chen, Yi-Chia Wei

**Affiliations:** 1 Department of Neurology, Linkou Chang Gung Memorial Hospital, Taoyuan, Taiwan; 2 College of Medicine, Chang Gung University, Taoyuan, Taiwan; 3 Department of Neurology, National Cheng Kung University Hospital, College of Medicine, National Cheng Kung University, Tainan, Taiwan; 4 Division of Endocrinology and Metabolism, Department of Internal Medicine, Linkou Chang Gung Memorial Hospital, Taoyuan, Taiwan; 5 Division of Hematology-Oncology, Department of Internal Medicine, Linkou Chang Gung Memorial Hospital, Taoyuan, Taiwan; 6 Biostatistical Consultation Center, Keelung Chang Gung Memorial Hospital, Keelung, Taiwan; 7 Division of Cardiology, Department of Internal Medicine, Keelung Chang Gung Memorial Hospital, Chang Gung University College of Medicine, Keelung, Taiwan; 8 Department of Neurology, Keelung Chang Gung Memorial Hospital, Keelung, Taiwan; 9 Institute of Neuroscience, National Yang Ming Chiao Tung University, Taipei, Taiwan; 10 Community Medicine Research Center, Keelung Chang Gung Memorial Hospital, Keelung, Taiwan; University of Cambridge, UNITED KINGDOM

## Abstract

**Background:**

Angiotensin receptor blockers (ARBs) may have protective effects against dementia occurrence in patients with hypertension (HTN). However, whether telmisartan, an ARB with peroxisome proliferator-activated receptor γ (PPAR-γ)–modulating effects, has additional benefits compared to other ARBs remains unclear.

**Methods and findings:**

Between 1997 and 2013, 2,166,944 type 2 diabetes mellitus (T2DM) patients were identified from the National Health Insurance Research Database of Taiwan. Patients with HTN using ARBs were included in the study. Patients with a history of stroke, traumatic brain injury, or dementia were excluded. Finally, 65,511 eligible patients were divided into 2 groups: the telmisartan group and the non-telmisartan ARB group. Propensity score matching (1:4) was used to balance the distribution of baseline characteristics and medications. The primary outcome was the diagnosis of dementia. The secondary outcomes included the diagnosis of Alzheimer disease and occurrence of symptomatic ischemic stroke (IS), any IS, and all-cause mortality. The risks between groups were compared using a Cox proportional hazard model. Statistical significance was set at *p* < 0.05. There were 2,280 and 9,120 patients in the telmisartan and non-telmisartan ARB groups, respectively. Patients in the telmisartan group had a lower risk of dementia diagnosis (telmisartan versus non-telmisartan ARBs: 2.19% versus 3.20%; HR, 0.72; 95% CI, 0.53 to 0.97; *p* = 0.030). They also had lower risk of dementia diagnosis with IS as a competing risk (subdistribution HR, 0.70; 95% CI, 0.51 to 0.95; *p* = 0.022) and with all-cause mortality as a competing risk (subdistribution HR, 0.71; 95% CI, 0.53 to 0.97; *p* = 0.029). In addition, the telmisartan users had a lower risk of any IS (6.84% versus 8.57%; HR, 0.79; 95% CI, 0.67 to 0.94; *p* = 0.008) during long-term follow-up. Study limitations included potential residual confounding by indication, interpretation of causal effects in an observational study, and bias caused by using diagnostic and medication codes to represent real clinical data.

**Conclusions:**

The current study suggests that telmisartan use in hypertensive T2DM patients may be associated with a lower risk of dementia and any IS events in an East-Asian population.

## Abstract 2

Chi-Hung Liu and co-workers study associations between antihypertensive medication prescriptions and dementia risk in people with type 2 diabetes.

## Author summary

**Why was this study done?:**

- The drug of choice for antihypertensive treatment, and its association with dementia in patients with both type 2 diabetes mellitus (T2DM) and hypertension, is a valid question worthy of further investigations.
- Angiotensin receptor blockers (ARBs) modulate the renin–angiotensin–aldosterone system and potentially associate with cognitive preservation, but internal comparisons of the cognitive protective effects of different ARBs have not previously been performed.
- Telmisartan is the only ARB that, at a clinical dose, has agonistic effects on peroxisome proliferator-activated receptor γ (PPAR-γ), which maintains insulin sensitivity, reduces insulin resistance, and potentially has a protective impact on dementia.

**What did the researchers do and find?:**

- Using the electronic health records of the National Health Insurance Research Database of Taiwan between 1997 and 2013, we studied whether telmisartan was associated with a lower incidence of dementia compared to other ARBs in patients with T2DM and hypertension.
- Using telmisartan was associated with a lower risk of dementia diagnosis compared to using non-telmisartan ARBs (2.19% versus 3.20%).

**What do these findings mean?:**

- Telmisartan use in hypertensive T2DM patients may be associated with a lower risk of dementia and any ischemic stroke events in an East-Asian population.
- Further work will be needed to investigate the observed association, owing to the limitations inherent in this observational study.

## Introduction

Hypertension (HTN) is a major risk factor for stroke and dementia [1,2]. Stroke can cause brain damage and result in cognitive impairment [3]. Furthermore, chronic HTN can induce alterations in the blood–brain barrier (BBB), vascular remodeling, and a reduction in regional cerebral flow [4], which can then lead to cognitive impairment in addition to the occurrence of stroke. The risk of dementia is also higher in patients with type 2 diabetes mellitus (T2DM), possibly due to vascular changes, alterations in glucose metabolism, and insulin signaling possibly resulting in neurodegeneration [5]. In T2DM patients with HTN, atherosclerosis, arterial remodeling, vascular inflammation, and endothelial dysfunction can progress more aggressively [6]. Patients with the coexistence of T2DM and HTN have also been reported to be more vulnerable to stroke and dementia [7,8], and therefore blood pressure (BP) control is an important issue in these patients.

Adequate HTN control has been shown to lower the risk of dementia [3,9]. In addition to the BP control goals of hypertensive medications, the class effect of antihypertensive medications in the prevention of stroke and dementia in HTN patients is also an important issue [10,11]. According to the American Diabetes Association guideline on cardiovascular disease management in diabetes patients, angiotensin receptor blockers (ARBs) or angiotensin converting enzyme inhibitors (ACEIs) are recommended for treatment of HTN in patients with T2DM for renal protection [12]. In addition, the effects of antihypertensive medications on the renin–angiotensin–aldosterone system (RAAS), insulin resistance (IR), and anti-inflammation can have further impacts in addition to the BP-lowering effect in these patients [6,13,14]. ARBs have RAAS-modulating effects and may confer pleiotropic protection on cognition [15]. IR may accelerate atherosclerosis, cause endothelial dysfunction, and impair cerebrovascular reserves [16,17]. Peroxisome proliferator-activated receptor γ (PPAR-γ) mediates the maintenance of whole-body insulin sensitivity [18]. A Cochrane review showed that PPAR-γ agonists could improve insulin sensitivity and decrease IR, and therefore reduce the incidence of recurrent stroke and cardiovascular death [19]. Previous studies have also demonstrated the protective effect of PPAR-γ agonists on dementia [20–22]. Among ARBs, telmisartan is the only one that has PPAR-γ-modulating activity under a clinical dose, and thus may improve IR [15,23–26]. However, whether the use of telmisartan, compared to other ARBs, is associated with better clinical cognitive and vascular outcomes in T2DM and HTN patients remains unknown. This study aimed to compare the long-term outcomes of telmisartan use with use of other ARBs in East-Asian T2DM patients with HTN.

## Methods

### Data source and patient identification

**Fig 1:**
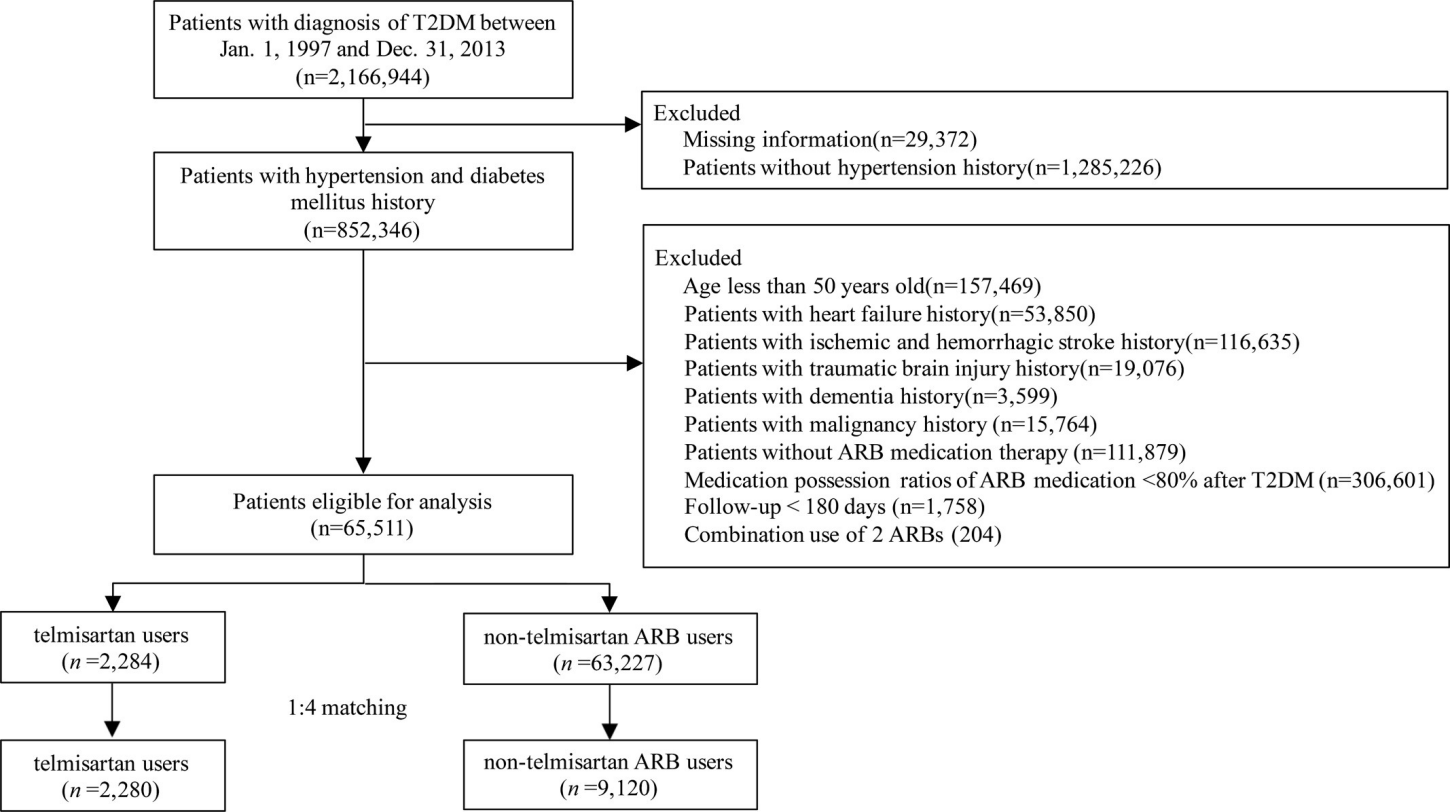
Flow chart for the inclusion of study patients. Patients with hypertension and T2DM were enrolled after the relevant exclusion criteria were applied. The patients were further divided into telmisartan and non-telmisartan ARB groups according to their prescribed antihypertensive drug. ARB, angiotensin receptor blocker; T2DM, type 2 diabetes mellitus.

This nationwide cohort study included all patients registered in the National Health Insurance Research Database (NHIRD), which contains claims data from the National Health Insurance (NHI) program in Taiwan. International Classification of Diseases, 9th Revision, Clinical Modification (ICD-9-CM) codes were used for the registration of all diagnoses, and the NHIRD is routinely monitored. Patients with T2DM were initially identified using the ICD-9-CM 250.XX diagnostic code between 1 January 1997 and 31 December 2013 [20]. Patients with missing information in the NHIRD were few and were not included. The current study focused on antihypertensive treatment, and therefore patients without a history of HTN were not included (Fig 1). To reduce the class effect of different antihypertensive drug categories, patients not receiving long-term ARB-based antihypertensive treatment were excluded. We also excluded patients with an ARB medication possession ratio < 80% after T2DM to achieve a sample with better drug compliance. This study aimed to study the impact of telmisartan on cognitive outcomes, so we also excluded patients with a history of stroke, traumatic brain injury, or dementia at enrollment. Patients with a history of heart failure and malignancy were excluded due to a higher risk of mortality. Both HTN and T2DM control at midlife are linked to preventing the later development of dementia [27,28]. Stroke is also an important risk factor for dementia, but the association between stroke and HTN could be less relevant in young stroke patients [29]. Therefore, patients aged <50 years at enrollment were excluded based on epidemiological investigation of dementia and clinical judgment of medical-comorbidity-related risk of developing dementia [30]. The detailed exclusion criteria are shown in Fig 1. The diagnostic codes for HTN and T2DM were validated in a previous NHIRD study [31]. To avoid misclassification bias due to coding errors, the included patients had to meet both the diagnosis and medication requirements. This study was approved by the ethics institutional review board of our hospital (approval number 202001445B1). Informed consent was waived since the data were anonymous. The RECORD checklist states the information of this observational study (S1 RECORD Checklist).

### Exposure to study drugs

We used a “pseudo-placebo” comparison group rather than an active comparator design. We divided the eligible patients into 2 groups based on the ARB they received during the 6-month exposure window after the index date: telmisartan or non-telmisartan ARBs. We extracted data on medications from outpatient claims data or pharmacy refills for chronic illnesses. The patients were defined as being ARB users if telmisartan or another ARB was prescribed continuously for 6 months (or more) at outpatient visits or as pharmacy refills. We excluded patients from the non-telmisartan ARB group if they had taken telmisartan for even 1 day during the 6-month exposure period, to ensure there was consistent use of the study drugs in the 2 groups. In addition, to assess adherence to the medication, we calculated the medication possession ratio as the number of days that the medication was prescribed divided by the number of days during the 6-month time period after the index date. An ARB medication possession ratio ≥ 80% was required for the patients in both study groups. Blood sugar and BP levels were not available in the NHIRD, and therefore we adjusted for the types and average numbers of oral antidiabetic drugs (OADs) and antihypertensive medications to mitigate the bias related to different levels of blood sugar and BP [32]. Cohort entry (index date) was defined as when the patient was diagnosed with T2DM and HTN and prescribed with antihypertensive and antidiabetic medications.

### Covariates

The covariates were age, sex, comorbidities (atrial fibrillation [AF], myocardial infarction [MI], chronic obstructive pulmonary disease, chronic kidney disease [CKD], dialysis, coronary artery disease [CAD], dyslipidemia, severe hypoglycemia, hypothyroidism, hyperthyroidism, depression, syphilis, and Charlson Comorbidity Index score), other antihypertensive medications (alpha blockers; diuretics including thiazide, loop diuretics, and spironolactone; beta blockers; and calcium channel blockers), insulin, OADs (dipeptidyl peptidase–4 inhibitors, secretagogue, alpha glucosidase, biguanide, pioglitazone, and sulfonylurea), and other medications (anticoagulants, fibrates, clopidogrel, statins, aspirin, and benzodiazepines). We extracted the patients’ baseline characteristics from the database, and obtained their medical records before the index date to track any history of major comorbidities. The comorbidities were defined if the patients had at least 1 inpatient diagnosis or 2 outpatient diagnoses of a given disease in the previous year. We used the NHI’s catastrophic illness certificate database to identify a history of malignancy or dialysis. Previous MI and stroke were identified by any inpatient diagnosis before the index date, which was tracked up to 1997. Validation studies of the diagnostic codes for these events and comorbidities have been performed previously (S1 Table) [33]. Patients’ overall systemic health was represented by Charlson Comorbidity Index score [34]. We also recorded the use of non-ARB medications via Taiwan NHI reimbursement and Anatomical Therapeutic Chemical codes from the claims data of outpatient visits and pharmacy refills within 6 months after the index date. S2 Table provides the Anatomical Therapeutic Chemical codes used for the drugs.

### Outcome measurement

The primary outcome was the diagnosis of dementia. The secondary outcomes included the diagnosis of Alzheimer disease (AD) and the occurrence of symptomatic ischemic stroke (IS), any IS, and all-cause mortality. The diagnostic codes for dementia were recorded by a neurologist and/or a psychiatrist at a minimum of 2 outpatient visits or 1 inpatient hospitalization (S1 Table) [35]. The diagnosis of AD was confirmed according to an ICD-9-CM code of 331.0 during the follow-up period. The Taiwan NHI regulations require confirmation of these diagnoses by a neurologist or a psychiatrist using the Mini‐Mental State Examination or the Clinical Dementia Rating scale, indicating good diagnostic accuracy of dementia and AD [35]. The occurrence of symptomatic IS was confirmed when patients were admitted to hospital during the follow-up period, primarily because of IS (S1 Table) [32]. All IS was confirmed when the patients were coded with IS during the follow-up period, including patients with minor symptomatic IS, those without hospitalization, and those with incidentally found silent infarctions identified during brain imaging studies. The ICD-9-CM diagnostic codes for dementia, AD, and IS have been validated in previous NHIRD studies [36,37]. We also identified the cause of death according to the registry data of the NHIRD, with the same definition as other registry studies [32]. The patients were followed up after cohort entry until an incident diagnosis of dementia or IS, end of registration with the NHI program, death, or 31 December 2013, whichever occurred first.

### Statistical analysis

We used propensity score matching (PSM) to balance the distribution of baseline characteristics, and the number of OADs and antihypertensive drug classes used, between the study groups. The propensity score indicated the predicted probability of being in the telmisartan group, given the values of the selected covariates. The covariates used to calculate the propensity score included the variables listed in Table 1, where the follow-up year was replaced with the index date. We did not allow any replacement after matching, and the matching order was random. To increase statistical power, a 1:4 matching ratio was used. We checked the quality of matching by means of the absolute standardized difference (ASTD) between the groups after matching. An absolute value < 0.1 was considered to indicate a nonsubstantial difference between the groups.

**Table 1:** Characteristics of the study patients with and without use of telmisartan after propensity score matching.

Characteristics	Telmisartan, *n* = 2,280	Non-telmisartan ARB, *n* = 9,120	ASTD
Age, years	62.4 ± 8.58	62.38 ± 8.54	0.002
Age group			
<65 years	1,482 (65.00%)	5,899 (64.68%)	0.007
65–74 years	567 (24.87%)	2,357 (25.84%)	0.022
≥75 years	231 (10.13%)	864 (9.47%)	0.022
Male	1,160 (50.88%)	4,640 (50.88%)	0.000
Comorbidities			
Atrial fibrillation	32 (1.40%)	164 (1.80%)	0.032
Myocardial infarction	38 (1.67%)	227 (2.49%)	0.057
Chronic obstructive pulmonary disease	718 (31.49%)	2,700 (29.61%)	0.041
Chronic kidney disease	109 (4.78%)	595 (6.52%)	0.075
Dialysis	51 (2.24%)	249 (2.73%)	0.031
Coronary artery disease	773 (33.90%)	3097 (33.96%)	0.001
Dyslipidemia	1,359 (59.61%)	5,208 (57.11%)	0.051
Severe hypoglycemia	2 (0.09%)	2 (0.02%)	0.030
Hypothyroidism	3 (0.13%)	17 (0.19%)	0.015
Hyperthyroidism	52 (2.28%)	226 (2.48%)	0.013
Depression	110 (4.82%)	450 (4.93%)	0.005
Syphilis	5 (0.22%)	18 (0.20%)	0.004
CCI total score	1.29 ± 1.31	1.32 ± 1.35	0.023
Antihypertensive agents			
Alpha blocker	899 (39.43%)	3,605 (39.53%)	0.002
Diuretic (thiazide, loop diuretic, spironolactone)	783 (34.34%)	3,124 (34.25%)	0.002
Beta blocker	1,772 (77.72%)	7,081 (77.64%)	0.002
CCB	2,012 (88.25%)	8,079 (88.59%)	0.011
Number of antihypertension drugs	3.21 ± 1.75	3.22 ± 1.73	0.006
Antidiabetic agents			
Insulin	85 (3.73%)	311 (3.41%)	0.017
DPP4i	44 (1.93%)	137 (1.50%)	0.033
Secretagogue (glinide)	84 (3.68%)	324 (3.55%)	0.007
Alpha glucosidase	101 (4.43%)	378 (4.14%)	0.014
Biguanide (metformin)	913 (40.04%)	3,687 (40.43%)	0.008
Sulfonylurea	670 (29.39%)	2,633 (28.87%)	0.011
Pioglitazone	296 (12.98%)	1,064 (11.67%)	0.040
Number of antidiabetic drugs	0.83 ± 0.84	0.82 ± 0.83	0.012
Other medications			
Anticoagulant	14 (0.61%)	50 (0.55%)	0.008
Fibrates	566 (24.82%)	2,326 (25.50%)	0.016
Clopidogrel	85 (3.73%)	325 (3.56%)	0.009
Statin	1,056 (46.32%)	4,174 (45.77%)	0.011
Aspirin	1,094 (47.98%)	4,380 (48.03%)	0.001
Benzodiazepine	1,784 (78.25%)	7,278 (79.80%)	0.038
Follow-up, years	4.91 ± 2.82	4.81 ± 3.38	0.032
Propensity score	0.038 ± 0.01	0.038 ± 0.01	0.000

ARB, angiotensin receptor blocker; ASTD, absolute standardized difference; CCB, calcium channel blocker; CCI, Charlson Comorbidity Index; DPP4i, dipeptidyl peptidase–4 inhibitor. Data given as mean ± standard deviation or *n* (%). An ASTD < 0.1 was considered a nonsubstantial difference between the groups.

It is noted that this study did not have a formal prespecified analysis plan. As for the time-to-event outcomes (i.e., symptomatic IS, any IS, and all-cause mortality), we used a Cox proportional hazard model to compare the risks between the study groups. We used the Fine and Gray subdistribution hazard model, which considered IS as a competing risk, to compare the time-to-dementia outcome between the 2 groups. We considered the study group (telmisartan versus non-telmisartan ARBs) as the only explanatory variable in the survival analyses. In addition, we conducted another post hoc analysis considering all-cause mortality during follow-up as a competing risk. Finally, we conducted subgroup analyses for dementia diagnosis based on 13 prespecified subgroup variables, including age, sex, CAD, CKD, chronic obstructive pulmonary disease, dyslipidemia, AF, number of antihypertensive agents, insulin use, aspirin use, pioglitazone use, statin use, and chronic benzodiazepine use. We used SAS version 9.4 (SAS Institute, Cary, NC, US) to perform all statistical analyses. Statistical significance was set at *p* < 0.05. Except for the additional analysis considering all-cause mortality as a competing risk, the other statistical analyses in this study were predefined.

## Results

### Study patients

Between 1 January 1997 and 31 December 2013, a total of 2,166,944 patients with a diagnosis of T2DM were recorded in the NHIRD. A total of 1,314,598 patients, including those who had missing information (29,372) and those who did not have a history of HTN (1,285,226), were excluded. Then, 111,879 patients who did not receive any ARB and 306,601 patients who had an ARB medication possession ratio < 80% were further excluded. In addition, patients who were aged < 50 years at enrollment (157,469) and those with a history of heart failure (53,850), previous stroke (116,635), previous traumatic brain injury (19,076), and dementia at enrollment (3,599) were also excluded. In total, 65,511 patients with T2DM and HTN were confirmed as eligible for analysis within the study based on the inclusion/exclusion criteria (Fig 1).

### Baseline characteristics

Before PSM, there were 2,284 patients in the telmisartan group and 63,227 patients in the non-telmisartan ARB group. The patients in the telmisartan group were younger; had a higher prevalence of dyslipidemia, CAD, and chronic obstructive pulmonary disease; and had higher Charlson Comorbidity Index scores. The patients in the telmisartan group also had a lower prevalence of CKD, lower average number of antidiabetic drugs, and a shorter follow-up duration (S3 Table). After PSM, there were 2,280 and 9,120 patients in the telmisartan and non-telmisartan ARB groups, respectively (Fig 1). All baseline characteristics and medications were well balanced between the 2 groups (Table 1).

### Primary outcome

**Fig 2:**
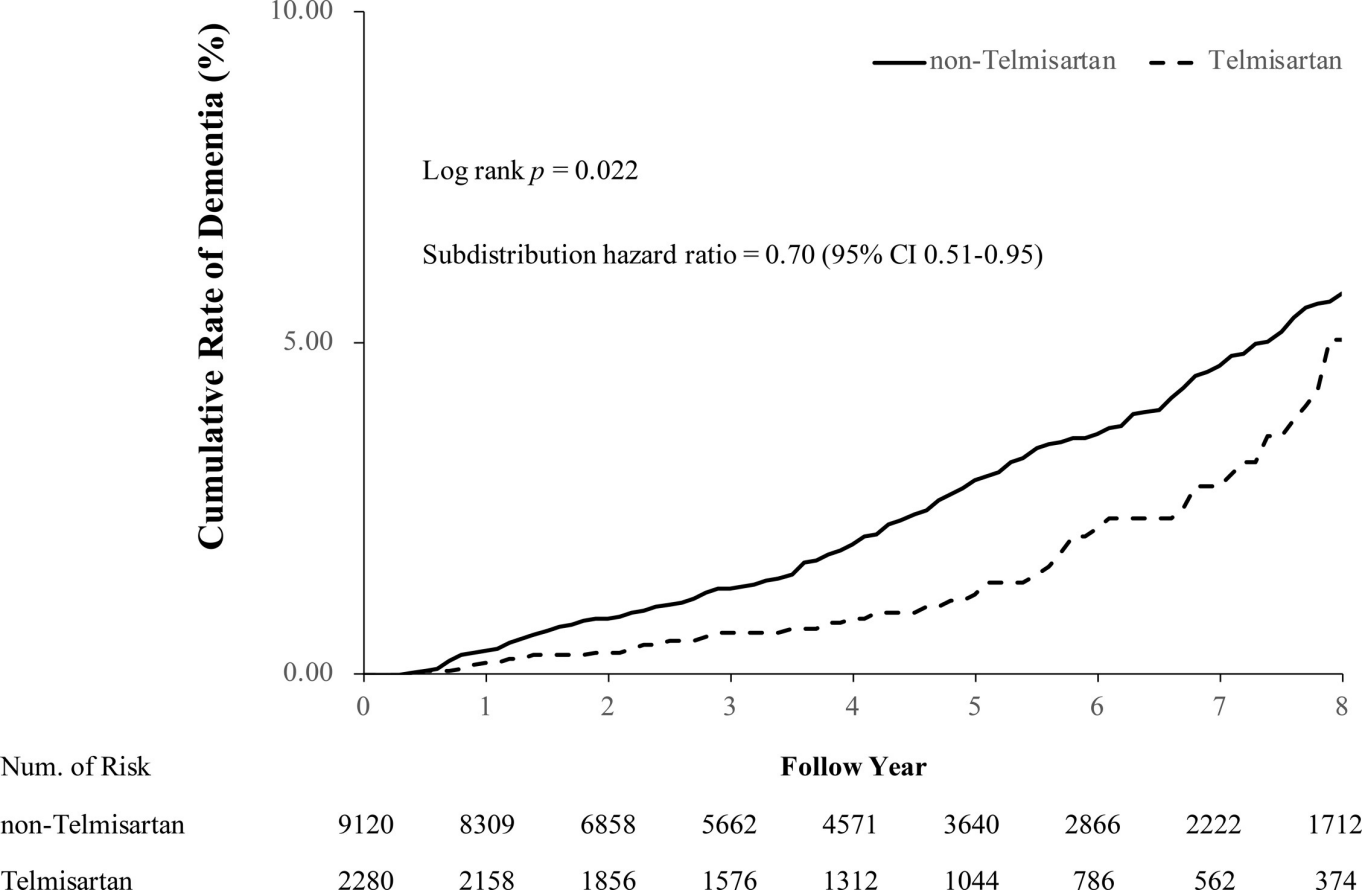
Comparison of the cumulative incidence of dementia between telmisartan and non-telmisartan angiotensin receptor blocker groups in the propensity-score-matched cohort. The curves show a lower risk of dementia diagnosis in the telmisartan group. The subdistribution hazard ratio considered ischemic stroke as a competing risk. The plot was truncated at the 8th year. CI, confidence interval.

The follow-up duration between the index date and the first outcome exposure was similar between the 2 groups (telmisartan versus non-telmisartan ARBs: 4.91 ± 2.82 versus 4.81 ± 3.38 years; ASTD = 0.032) after PSM (Table 1). The primary outcome was compared between the 2 study groups. Compared to the non-telmisartan ARB group, the telmisartan group had a lower risk of dementia diagnosis (telmisartan versus non-telmisartan ARBs: 2.19% versus 3.20%; hazard ratio [HR], 0.72; 95% confidence interval [CI], 0.53 to 0.97; *p* = 0.030; Table 2). As stroke occurrence may confound the occurrence of dementia, we further analyzed the dementia outcome for the patients without a stroke diagnosis prior to their dementia diagnosis. The telmisartan group still had a lower risk of dementia diagnosis (subdistribution HR, 0.70; 95% CI, 0.51 to 0.95; *p* = 0.022; Table 2) compared with the non-telmisartan ARB group, as shown in the cumulative incidence plot (Fig 2). In addition, the telmisartan group had a lower risk of dementia diagnosis when considering all-cause mortality as a competing factor (subdistribution HR, 0.71; 95% CI, 0.53 to 0.97; *p* = 0.029; Table 2).

**Table 2:** Occurrence of primary and secondary outcomes of patients with and without use of telmisartan.

Outcome	Number (%) with outcome		Telmisartan versus non-telmisartan ARBs	
	Telmisartan, *n* = 2,280	Non-telmisartan ARB, *n* = 9,120		
			HR (95% CI)	*p*-Value
Primary outcome—dementia				
Diagnosis of dementia	50 (2.19)	292 (3.20)	0.72 (0.53, 0.97)	0.030
Subdistribution HR considering ischemic stroke as competing risk			0.70 (0.51, 0.95)	0.022
Subdistribution HR considering all-cause mortality as competing risk			0.71 (0.53, 0.97)	0.029
Secondary outcomes				
Alzheimer disease	3 (0.13)	21 (0.23)	0.59 (0.18, 2.00)	0.400
Symptomatic ischemic stroke	35 (1.54)	182 (2.00)	0.79 (0.55, 1.14)	0.207
Any ischemic stroke	156 (6.84)	782 (8.57)	0.79 (0.67, 0.94)	0.008
All-cause mortality	13 (0.57)	63 (0.69)	0.99 (0.54, 1.81)	0.966

ARB, angiotensin receptor blocker; CI, confidence interval; HR, hazard ratio. Statistical significance was set at *p* < 0.05.

### Secondary outcomes

**Fig 3:**
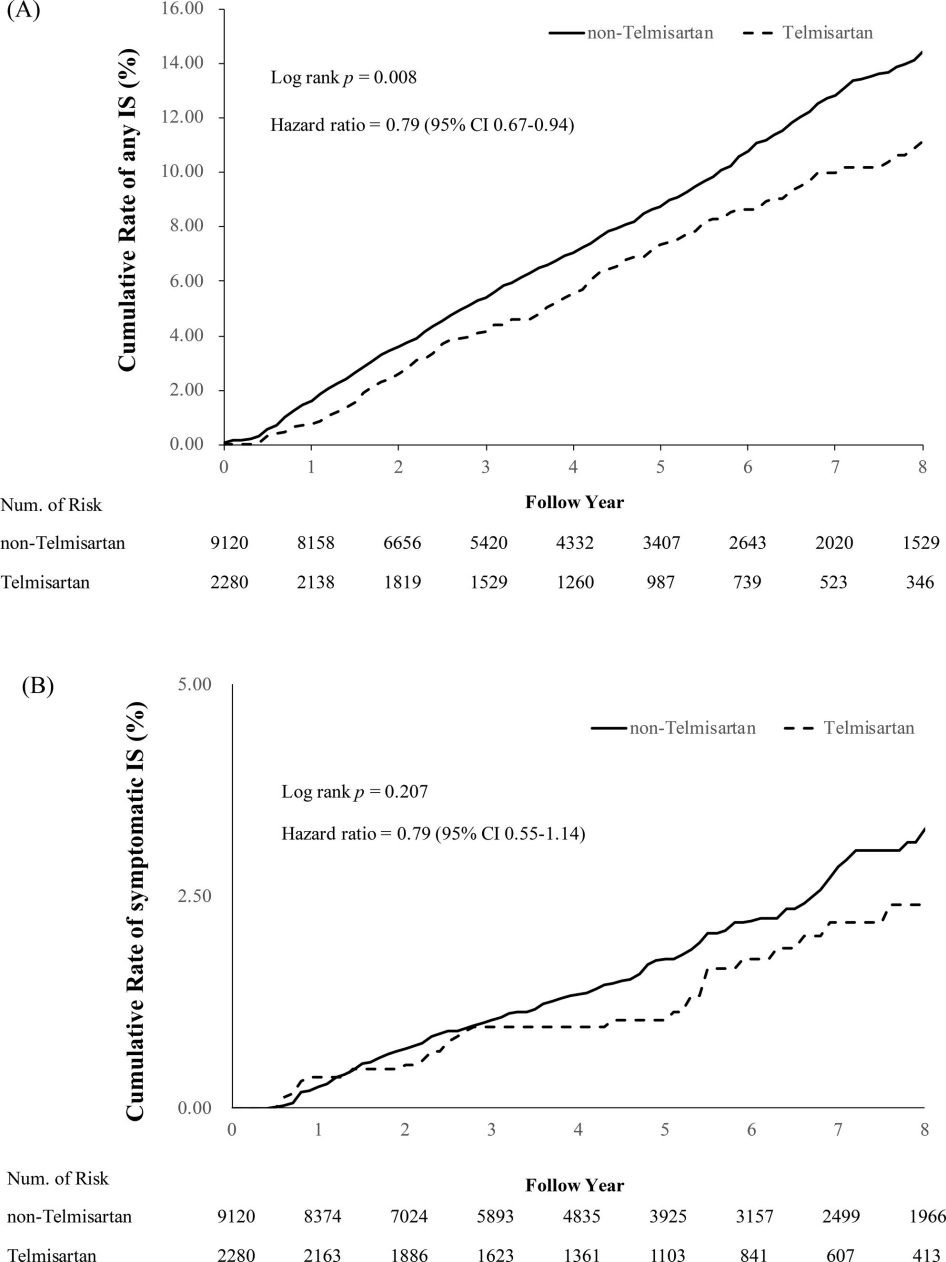
Comparison of the cumulative incidence of IS between the telmisartan and non-telmisartan ARB groups in the propensity-score-matched cohort. The curves show lower risks of any IS (A) but not of new symptomatic IS (B) in the telmisartan group. The plot was truncated at the 8th year. CI, confidence interval; IS, ischemic stroke.

Compared with the non-telmisartan ARB group, the telmisartan group had a lower risk of any IS (telmisartan versus non-telmisartan ARBs: 6.84% versus 8.57%; HR, 0.79; 95% CI, 0.67 to 0.94; *p* = 0.008; Fig 3A). The risks of symptomatic IS (telmisartan versus non-telmisartan ARBs: 1.54% versus 2.00%; HR, 0.79; 95% CI, 0.55 to 1.14; *p* = 0.207; Fig 3B), all-cause mortality (telmisartan versus non-telmisartan ARBs: 0.57% versus 0.69%; HR, 0.99; 95% CI, 0.54 to 1.81; *p* = 0.966), and AD (telmisartan versus non-telmisartan ARBs: 0.13% versus 0.23%; HR, 0.59; 95% CI, 0.18 to 2.00; *p* = 0.400) were not significantly different between the 2 groups at the end of the follow-up period (Table 2).

### Subgroup analyses for the risk of dementia among patients with various baseline characteristics and comorbidities

Subgroup analyses defined by various baseline characteristics did not reveal significant interactions for the observed effects of telmisartan (S1 Fig).

## Discussion

This nationwide cohort study showed that the long-term use of telmisartan, compared with other ARBs, in T2DM patients with HTN may be associated with lower risk of dementia diagnosis, after adjusting for IS occurrence or all-cause mortality as a competing factor. Telmisartan use was also associated with fewer IS events. HTN has been associated with a reduction in cognitive performance in middle-aged and older adults [38]. A previous study also showed a U-shaped association between BP and cognition [39]. The mean age of our enrolled patients was younger than in some previous studies [10], which highlights the particularly significant beneficial effects of BP control on cognition in midlife adults [4,40–42]. There was no significant interaction between different ages in the subgroup analyses. It is possible that the lower number of patients aged 65 years or older in our study could be one of the causes of this result. This may limit the generalizability of our findings to a wider age population. Regarding the protective effects of different classes of anti-HTN medication on cognitive function, to date, the drug of choice for antihypertensive treatment, and its association with dementia occurrence, remains controversial. Recent meta-analyses have not identified a specific class of antihypertensive medication to be more effective than others in lowering the risk of dementia [10,11]. However, comparisons between different ARBs were not evaluated in those studies. Moreover, the number of ARB users was low, and adherence to BP treatment was unavailable in those selected cohorts [10]. In addition, compared to our work, the enrolled patients in those studies were older and had higher frequency of cardiovascular diseases [10]. These factors may have led to a higher risk of mortality, which is a major competing risk for the study results. The previous studies also focused on the general population rather than specifically on T2DM patients. The impact of HTN treatment on the risk of dementia occurrence in T2DM patients is a valid question that is worthy of further investigation [4,43]. Our data suggest an association between a lower occurrence of dementia diagnosis and telmisartan use in T2DM patients. This could guide further clinical trials to verify our findings.

Telmisartan is the only ARB with PPAR-γ-modulating effects when given at a clinical dose [44]. PPAR-γ may regulate metabolism, improve IR, and have neuroprotective effects [18,45]. IR may impair endothelial nitric oxide synthase and induce the excitatory effect of hyperglycemia on RAAS, which could have a negative impact on cardiovascular disease [46]. Hyperinsulinemia is also a known risk factor for dementia and accelerated cognitive decline [47]. PPAR-γ has been shown to affect amyloid-β toxicity, improve mitochondrial dysfunction, and reduce inflammation [48], and pioglitazone has been shown to have protective effects against stroke and dementia occurrence [20,49–51]. Compared to pioglitazone, the PPAR-γ-modulating effects of telmisartan are lower [23,44]; however, they have been demonstrated to be sufficient to improve IR [24,25]. The frequency of AD diagnosis was similar between the 2 study groups in the present study, and the subgroup analysis did not show a stronger protective effect for concurrent pioglitazone use. However, the number of patients who were newly diagnosed with AD or used a combination of telmisartan and pioglitazone may be insufficient to demonstrate clinical effects.

Telmisartan has been shown to increase the secretion of adiponectin [52]. Although the correlation between changes in adiponectin levels and clinical outcomes remains controversial [53], elevated adiponectin levels may be correlated with increased insulin sensitivity, and anti-inflammatory, anti-atherosclerotic, and anti-thrombotic effects [54,55]. Telmisartan has not shown significant stroke preventive effects in previous trials [56,57]. However, one previous real-world study revealed that in T2DM patients after IS, the use of telmisartan in addition to pioglitazone tended to reduce IS recurrence. In our study, the telmisartan users did not have lower symptomatic IS occurrence; however, they had a significantly lower incidence of any IS event, which may reduce the risk of vascular dementia [33]. Telmisartan may also protect against cognitive decline by means of tropomyosin-related kinase B and brain-derived neurotrophic factor up-regulation in the hippocampus [58]. Mice models have also revealed that telmisartan has a protective effect against cognitive impairment [59,60]. Although our results provide potential clinical evidence to show an association of telmisartan with dementia risk reduction, further studies are warranted to clarify the causal relationship of such therapy and the underlying mechanisms of action.

RAAS inhibition may play a role in dementia-related cognitive decline [61,62]. RAAS inhibitors may influence the amyloid cascade, and central RAAS modulation may have an effect on cognition [63]. Centrally acting ACEIs and ARBs were demonstrated to potentially prevent cognitive impairment in an animal model [61]. Compared to ACEIs, ARBs can further lower the risk of dementia due to their unique effects on angiotensin type I receptors and angiotensin IV signaling [63]. The Ongoing Telmisartan Alone and in Combination with Ramipril Global Endpoint Trial showed that telmisartan users might have marginally lower risks of stroke or cognitive impairment compared to ramipril users [64]. Telmisartan has also been shown to have sufficient BP-lowering potency [65], to have adequate BBB penetration, and to reduce BP variability [63,66,67], all of which can influence the cognitive protective effects [68,69]. BP variability is a risk factor for leukoaraiosis and silent infarction progression [68,70], which have also been shown to be important factors in the development of dementia [71]. The effect of appropriate BP control on reducing the risk of dementia occurrence is uncertain [72]. The Systolic Blood Pressure Intervention Trial–MIND study only demonstrated inconclusive results for the effect of intensive BP control on dementia [73]. In our study, the telmisartan users also had a lower incidence of any IS. The patients registered as having any IS may have had minor strokes or silent infarctions, which were observed when the patients were receiving brain imaging for dementia surveys. This could also be a possible explanation for the lower incidence of dementia in the telmisartan group.

There are some limitations to the present study. First, the homeostasis model assessment of the IR index, blood sugar, and BP levels were not recorded in the claims database. Moreover, patients who developed dementia without the correct diagnostic coding or outpatient visits or who developed IS without hospitalization were not registered in the data. This may have caused selection bias and could have affected our interpretation of the data. To mitigate these selection biases and residual or unmeasured confounders, we adjusted for the number of antihypertensive and antidiabetic drugs used at baseline and used the Charlson Comorbidity Index to balance the baseline characteristics of the study groups. However, some comorbidities, such as obesity, could not be well documented in the claims data study. Compared to other ARBs, telmisartan could be a prior choice in patients with a high cardiovascular burden due to its cardioprotective effects [74]. This may raise the possibility of confounding by indication which the telmisartan users are more vulnerable to developing CAD and stroke events. Before PSM, the frequencies of MI and AF were not different between the telmisartan and non-telmisartan ARB groups. However, the telmisartan users were younger, had lower frequencies of female and CKD patients, and had higher frequencies of receiving preventive medication (aspirin, clopidogrel, or statins) for cerebrovascular disease (S3 Table). The incidence rate of dementia is higher in female and CKD patients [75,76]. Patients taking medications for stroke prevention may also have less burden of dementia occurrence [77]. These factors may generate a potential bias that the telmisartan users in our study could be less likely to have dementia due to these age and health profile differences. Although we used PSM to balance these differences, PSM can only relate to the data that exist and cannot fully address the gaps. The PSM in our study may still have been insufficient to serve as a proxy of disease control. A randomized controlled trial is warranted to provide a more conclusive answer.

Second, drug adherence, switching, or combinations may have confounded the study results. In this study, we estimated the patients’ drug adherence and compliance based on their prescription records, which could be biased with respect to the true medication-taking behavior. By year 8, there appeared to be a much smaller difference, even though it remained statistically significant (Fig 2). This could be related to switching or discontinuing the study ARBs. This neutral effect also occurred when the enrolled patients received longer follow-up in the Action to Control Cardiovascular Risk in Diabetes trial [78].

Third, the ICD-9-CM codes could have been incorrectly coded in the claims database. The use of electronic health records to confirm the diagnoses of dementia, AD, and IS may also have led to under- or misdiagnosis. However, in Taiwan, physicians and their institutions are penalized if their clinical coding violates clinical guidelines and consensus. According to NHI regulations, medical reimbursement specialists also review and inspect all insurance claims. Furthermore, the confirmation of dementia diagnosis is restricted to a neurologist or a psychiatrist after a thorough clinical follow-up and neuropsychiatry evaluations according to NHI regulations, which may reduce the bias that could have influenced our conclusions. However, data on the results of the Mini-Mental State Examination, years of education, and premorbid function were not available in the claims database. This may have confounded the accuracy and types of dementia diagnoses in our study and limited our ability to make generalizable conclusions.

Fourth, in this observational study, the causal effects of the study drugs should be interpreted with caution. The results of this observational study remain insufficient to give conclusive answers of a high evidence level. However, our findings may help to inspire future studies on dementia risk reduction in hypertensive T2DM patients.

Lastly, it is unclear whether the conclusions of this study can be generalized to other ethnicities.

### Conclusion

The current study suggests that the use of telmisartan in hypertensive T2DM patients may be associated with lower risks of dementia and any IS events in an East-Asian population. Further clinical trials and basic research are warranted to confirm the protective effects of telmisartan and to explore the possible underlying mechanisms of action.

## Supporting information

S1 RECORD Checklist (DOCX)

S1 Fig Subgroup analyses of dementia diagnosis. Statistical significance for the interaction of subgroup analyses was set at *p* < 0.05. CI, confidence interval; HR, hazard ratio.
(TIF)

S1 Table ICD-9-CM codes used for diagnosis in the current study. (DOCX)

S2 Table Anatomical Therapeutic Chemical (ATC) codes used for drugs in the current study. (DOCX)

S3 Table Characteristics of the study patients with and without the use of telmisartan before propensity score matching. (DOCX)

## Abbreviations

ACEI: angiotensin converting enzyme inhibitor
AD: Alzheimer disease
AF: atrial fibrillation
ARB: angiotensin receptor blocker
ASTD: absolute standardized difference
BBB: blood–brain barrier
BP: blood pressure
CAD: coronary artery disease
CI: confidence interval
CKD: chronic kidney disease
HR: hazard ratio
HTN: hypertension
ICD-9-CM: International Classification of Diseases, 9th Revision, Clinical Modification
IR: insulin resistance
IS: ischemic stroke
MI: myocardial infarction
NHI: National Health Insurance
NHIRD: National Health Insurance Research Database
OAD: oral antidiabetic drug
PPAR-γ: peroxisome proliferator-activated receptor γ
PSM: propensity score matching
RAAS: renin–angiotensin–aldosterone system
T2DM: type 2 diabetes mellitus
