# Supplementary figures and images for: Telmisartan use and risk of dementia in type 2 diabetes patients with hypertension: A population-based cohort study
Source: PLoS Med. 2021 Jul 19;18(7):e1003707. doi: 10.1371/journal.pmed.1003707 (PMC8289120; doi:10.1371/journal.pmed.1003707)

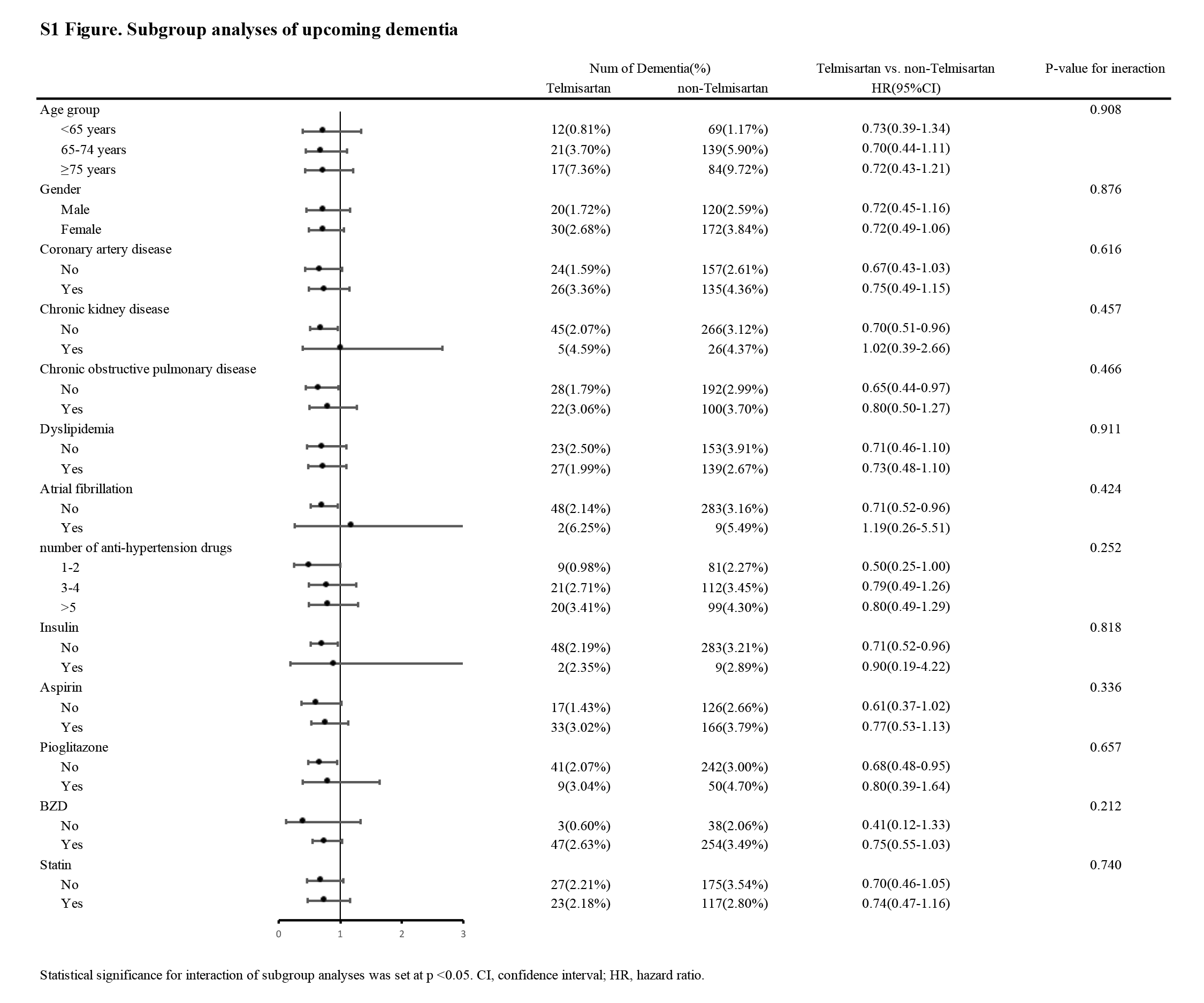

Supplement: S1 Fig — Statistical significance for the interaction of subgroup analyses was set at p < 0.05. CI, confidence interval; HR, hazard ratio. (TIF) [file pmed.1003707.s002.tif]
