# Supplementary material for: Telmisartan use and risk of dementia in type 2 diabetes patients with hypertension: A population-based cohort study
Source: PLoS Med. 2021 Jul 19;18(7):e1003707. doi: 10.1371/journal.pmed.1003707 (PMC8289120; doi:10.1371/journal.pmed.1003707)
Supplement: S1 Table — (DOCX) [file pmed.1003707.s003.docx]

**S1 Table.** ICD-9-CM code used for diagnosis in the current study

| Variable | Code |
| --- | --- |
| Ischemic stroke | 433.xx–435.xx, excluding 433.00, 433.10, 433.20, 433.30, 433.80, 433.90, 434.00, 434.10, 434.90 |
| Hypertension | 401.xx–405.xx and any anti-hypertension drugs |
| Diabetes mellitus | 250.xx and any oral hypoglycemic drugs and insulin |
| Heart failure | 428.xx |
| Any stroke | 430.xx–437.xx |
| Previous myocardial infarction | 410.xx, 412.xx |
| Coronary artery disease | 410.xx–414.xx |
| Chronic kidney disease | 580.xx–589.xx, 403.xx–404.xx, 016.0x, 095.4x, 236.9x, 250.4x, 274.1x, 442.1x, 447.3x, 440.1x, 572.4x, 642.1x, 646.2x, 753.1x, 283.11, 403.01, 404.02, 446.21 |
| Dialysis | 585.xx (Catastrophic illness certificate) |
| Chronic obstructive pulmonary disease | 491.xx, 492.xx, 496.xx |
| Atrial fibrillation | 427.31 |
| Dyslipidemia | 272.xx and any lipid-lowing agents |
| Malignancy | 140.xx–208.xx (Catastrophic illness certificate) |
| Cirrhosis | 571.2, 571.5, 571.6 |
| Acute myocardial infarction | 410.xx |
| Hyperthyroidism | 242.xx |
| Hypothyroidism | 244.9 |
| Depression | 296.2, 296.3, 300.4, 311 |
| Syphilis | 090-097 |
| Traumatic brain injury | 800-804, 850-854 |
| Dementia | 290.0-290.4, 294.1, 331.0-331.2, 331.82, 438.0 |

ICD-9-CM, International Classification of Diseases, Ninth Revision, Clinical Modification.
