# Supplementary material for: Telmisartan use and risk of dementia in type 2 diabetes patients with hypertension: A population-based cohort study
Source: PLoS Med. 2021 Jul 19;18(7):e1003707. doi: 10.1371/journal.pmed.1003707 (PMC8289120; doi:10.1371/journal.pmed.1003707)
Supplement: S2 Table — (DOCX) [file pmed.1003707.s004.docx]

**S2 Table.** Anatomical Therapeutic Chemical (ATC) codes used for drugs in the current study

| Medications | ATC code |
| --- | --- |
| **Study drugs** |  |
| ARB | C09 |
| Telmisartan | C09CA07, C09DB04, C09DA07 |
| **Anti-hypertensive drugs** |  |
| Telmisartan | C09CA07, C09DB04, C09DA07 |
| Alpha-blocker | C02 |
| Diuretics (Thiazide/Loop diuretics/Spironolactone) | C03AA03, 03AA06, C03AA91, C03AA07, C03CA01, C03CA02, C03DA01, C03EA01 |
| Beta-blocker | C07 |
| CCB | C08 |
| **Anti-diabetes mellitus drugs** |  |
| Insulin | A10A |
| DPP4i | A10BH |
| Secretagogue (Glinide) | A10BX02, A10BX03 |
| Alpha glucosidase | A10BF |
| Biguanide (Metformin) | A10BA |
| Sulfonylurea | A10BB |
| Pioglitazone | A10BG03, A10BD05 |
| **Other medications** |  |
| Anticoagulant | B01AA02, B01AA03, B01AE07, B01AF01, B01AF02, B01AF03 |
| Fibrate | C10AB01, C10AB02, C10AB03, C10AB04, C10AB05, C10AB06, C10AB09 |
| Clopidogrel | B01AC04, B01AC30 |
| Statin | C10AA |
| Aspirin | B01AC06 |
| Benzodiazepines | N05B, N05C |

ARB, angiotensin II receptor blockers; CCB, calcium channel blockers; DPP4i, dipeptidyl peptidase-4 inhibitor.
