# Supplementary material for: Telmisartan use and risk of dementia in type 2 diabetes patients with hypertension: A population-based cohort study
Source: PLoS Med. 2021 Jul 19;18(7):e1003707. doi: 10.1371/journal.pmed.1003707 (PMC8289120; doi:10.1371/journal.pmed.1003707)
Supplement: S3 Table — (DOCX) [file pmed.1003707.s005.docx]

**S3 Table.** Characteristics of the study patients with and without the use of telmisartan before propensity score matching

| Characteristics | Telmisartan  (*n* = 2,284) | Non-telmisartan ARBs  (*n* = 63,227) | P value |
| --- | --- | --- | --- |
| Age, years | 62.39±8.58 | 63.15±8.79 | <0.001 |
| Age group, n (%) |  |  | 0.001 |
| <65 years | 1486(65.10%) | 38723(61.24%) |  |
| 65-74 years | 567(24.82%) | 17361(27.46%) |  |
| ≥75 years | 231(10.11%) | 7143(11.30%) |  |
| Male, n (%) | 1164(50.96%) | 30025(47.49%) | 0.001 |
| Comorbidity, n (%) |  |  |  |
| Atrial fibrillation | 33(1.44%) | 1027(1.62%) | 0.504 |
| Myocardial infarction | 39(1.71%) | 1448(2.29%) | 0.073 |
| Chronic obstructive pulmonary disease | 720(31.52%) | 18356(29.03%) | 0.010 |
| Chronic kidney disease | 109(4.77%) | 3887(6.15%) | 0.007 |
| Dialysis | 51(2.23%) | 1618(2.56%) | 0.331 |
| Dyslipidemia | 1362(59.63%) | 33242(52.58%) | <0.001 |
| Severe hypoglycemia | 2(0.09%) | 27(0.04%) | 0.268 |
| Hypothyroidism | 3(0.13%) | 65(0.10%) | 0.677 |
| Hyperthyroidism | 52(2.28%) | 1347(2.13%) | 0.606 |
| Depression | 110(4.82%) | 2951(4.67%) | 0.724 |
| Syphilis | 5(0.22%) | 137(0.22%) | 0.821 |
| CCI total score | 1.29±1.31 | 1.25±1.33 | 0.154 |
| Anti-hypertensive agent, n (%) |  |  |  |
| Alpha-blocker | 900(39.40%) | 25448(40.25%) | 0.420 |
| Diuretics (Thiazide/Loop diuretics/Spironolactone) | 785(34.37%) | 21137(33.43%) | 0.350 |
| Beta-blocker | 1776(77.76%) | 48928(77.38%) | 0.675 |
| CCB | 2016(88.27%) | 55021(87.02%) | 0.082 |
| Average number of anti-hypertension drugs | 3.21±1.75 | 3.22±1.75 | 0.781 |
| Antidiabetic agent, n (%) |  |  |  |
| Insulin | 85(3.72%) | 2507(3.97%) | 0.558 |
| DPP4i | 47(2.06%) | 856(1.35%) | 0.008 |
| Secretagogue (Glinide) | 84(3.68%) | 1769(2.8%) | 0.017 |
| Alpha glucosidase | 104(4.55%) | 2158(3.41%) | 0.003 |
| Biguanide (Metformin) | 915(40.06%) | 27826(44.01%) | 0.001 |
| Sulfonylurea | 670(29.33%) | 24167(38.22%) | <0.001 |
| Average number of Antidiabetic drugs | 0.83±0.84 | 0.94±0.86 | <0.001 |
| Other medications, n (%) |  |  |  |
| Anticoagulant | 14(0.61%) | 392(0.62%) | 0.967 |
| Fibrate | 566(24.78%) | 16044(25.38%) | 0.521 |
| Clopidogrel | 87(3.81%) | 1938(3.07%) | 0.049 |
| Statin | 1060(46.41%) | 25233(39.91%) | <0.001 |
| Aspirin | 1098(48.07%) | 28515(45.10%) | 0.005 |
| Benzodiazepines | 1788(78.28%) | 50195(79.39%) | 0.200 |
| Follow-up years | 4.91±2.82 | 5.52±3.79 | <0.001 |

DM, diabetes mellitus; CCI, Charlson Comorbidity Index; CCB, calcium channel blockers; DPP4i, dipeptidyl peptidase-4 inhibitor; ASTD, absolute standardized difference.
